# Psychiatric training in perinatal mental health across Europe

**DOI:** 10.1007/s00737-022-01216-w

**Published:** 2022-03-03

**Authors:** Marisa Casanova Dias, Ekin Sönmez Güngör, Sean Naughton, Howard Ryland, Thomas Gargot, Mariana Pinto da Costa, Athanasios Kanellopoulos, Franziska Baessler, Livia De Picker

**Affiliations:** 1 MRC Centre for Neuropsychiatric Genetics and Genomics, National Centre for Mental Health, Cardiff University, Cardiff, Wales UK; 2 Section of Women’s Mental Health, Institute of Psychiatry, Psychology and Neurosciences, King’s College London, London, UK; 3 University of Health Sciences, Erenköy Mental Health and Neurological Diseases Training and Research Hospital, Istanbul, Turkey; 4 Specialist Perinatal Mental Health Service, Rotunda Hospital, Dublin, Ireland; 5 Department of Psychiatry, University of Oxford, Oxford, UK; 6 Child and Adolescent Psychiatry Department, UMR U1253 « Imaging and Brain », iBrain, University Hospital of Tours, Tours, France; 7 South London and Maudsley NHS Foundation Trust, London, UK; 8 Institute of Psychiatry, Psychology and Neurosciences, King’s College London, London, UK; 9 Institute of Biomedical Sciences Abel Salazar, University of Porto, Porto, Portugal; 10 Center for Adolescent Medicine, First Department of Pediatrics, School of Medicine, National and Kapodistrian University of Athens, Aghia Sophia Children’s Hospital, Athens, Greece; 11 Centre for Psychosocial Medicine, Department of General Internal and Psychosomatic Medicine, Heidelberg University Hospital, Heidelberg, Germany; 12 Heidelberg Academy of Sciences and Humanities, Heidelberg, Germany; 13 Collaborative Antwerp Psychiatric Research Institute, University of Antwerp, Antwerp, Belgium; 14 University Psychiatric Hospital Campus Duffel, Antwerp, Belgium

**Keywords:** Perinatal mental health, Psychiatric training, Postgraduate training, European Federation of Psychiatric Trainees (EFPT)

## Abstract

Perinatal mental illness is associated with considerable maternal and infant morbidity and mortality. However, there are currently no specific guidelines on the standards and structure of postgraduate perinatal psychiatric training in Europe. We describe the characteristics of available and desired specialist perinatal psychiatry training from the perspective of European psychiatrists in training. An online survey was conducted among 34 national psychiatric trainee association representatives of the European Federation of Psychiatric Trainees (EFPT). Participants from the countries in which perinatal psychiatry training was available were invited to participate in in-depth follow-up interviews. Six countries out of 34 (18%) reported that specialist training in perinatal mental health was available (Finland, France, Germany, Ireland, Malta, and the UK). The nature of available training varied in duration, the supervision and assessment model employed, and the training scheme context. Of the 28 countries where specialist perinatal psychiatry training was unavailable, the majority of national representatives (22 countries, 76%) wanted specialist perinatal psychiatry training to be included in their national training curricula. There is a gap between the expected skills and the available training for psychiatrists to meet the mental healthcare needs of women in the perinatal period. Given the prevalence and impact of perinatal mental illness and the expressed desires of trainees themselves for specialist training, this finding should prompt urgent action.

**Supplementary Information:**

The online version contains supplementary material available at 10.1007/s00737-022-01216-w.

## Introduction

Pregnancy, childbirth, and the postpartum (the ‘perinatal period,’ conventionally defined as pregnancy to one year postpartum) is a high-risk time for the exacerbation of existing psychiatric illness and the development of new-onset conditions. Perinatal mental illness is associated with considerable maternal and foetal/infant morbidity and mortality (Howard et al. 2014; Jones et al. 2014). It can have a devastating effect on women, their families, and their child’s development. Therefore, timely action can significantly improve health outcomes (Casanova Dias et al. 2021; Stein et al. 2014).

Perinatal mental healthcare has been gaining momentum in Europe in recent years, with investment in new specialist services in some high-income countries (e.g. UK and Ireland) and mother-baby units elsewhere (Brockington et al. 2017; Howard and Khalifeh 2020). Providing high-quality perinatal mental healthcare requires clinicians from allied disciplines to possess adequate skills and ‘think family’. The development of clinical services and infrastructure should therefore be matched by the development of training programmes for clinicians to deliver these services adequately. The aim of postgraduate psychiatric training is to offer psychiatrists the necessary skills and preparation to practice independently. The quality of care provided will depend on the curriculum and training they receive (Casanova Dias et al. 2016). In Europe, the frameworks for training standards are issued by the European Union of Medical Specialists (UEMS, www.uemspsychiatry.org). The European Federation of Psychiatric Trainees (EFPT, www.efpt.eu), an umbrella organisation for national psychiatric trainee associations, collaborates with UEMS to produce statements that reflect trainees’ recommendations for high-quality training. A minimum of 5 years of training is recommended, with practical experience in different areas of psychiatric practice, including exposure to psychiatric conditions throughout the life span across Europe. However, there are currently no European recommendations specifically pertaining to perinatal psychiatry, and the situation on the ground still varies widely between countries (Baessler et al. 2015, 2021; Kuzman et al. 2012). For instance, whilst 80% of countries stipulate a placement in a non-psychiatric specialty such as internal medicine or neurology, only 40% do so for substance abuse and 26% for old age psychiatry (Baessler et al. 2021).

In England, a competency framework was commissioned to inform perinatal training provision for all professions and standardise competencies (The Tavistock and Portman NHS Foundation Trust 2018). The need for such standardised cross-disciplinarily frameworks is likely a reflection of the similar variation in mental healthcare training of allied healthcare professionals.

The objective of our study is to describe the characteristics of available and desired postgraduate perinatal psychiatry training across Europe from the perspective of psychiatrists in training.

## Materials and methods

### Data collection

This cross-sectional observational study was conducted in 2016. It was part of a broader online survey of postgraduate training that EFPT conducts annually in all member countries. Online questionnaire with multiple-choice and free-text questions was sent to the representatives of each national psychiatric trainee association or, if unavailable, to trainees with comparable knowledge (see Supplement). The questionnaire inquired about the details of the training in perinatal psychiatry (e.g., availability, whether optional or mandatory, duration) in addition to general aspects of training structure and content. Where training was reported as not available, respondents’ opinions were solicited about their preferences for such training and how it could be delivered in their respective countries. Where training was available, online semi-structured interviews were conducted via video call and email communication with trainee representatives. Further information was gathered about the setting, conditions, teams, assessment methodology, supervision, and research opportunities available. Personal views on the training received were also solicited. Informed consent was obtained from all country representatives and interview participants.

### Data analysis

Descriptive statistics of online survey respondents are reported, including the profile of available training and expressed preferences for development. The oral interviews were transcribed, and information about the characteristics of perinatal mental health training was extracted and categorised to provide additional descriptive analyses.

## Results

### Available specialist training in perinatal psychiatry across Europe

**Fig. 1:**
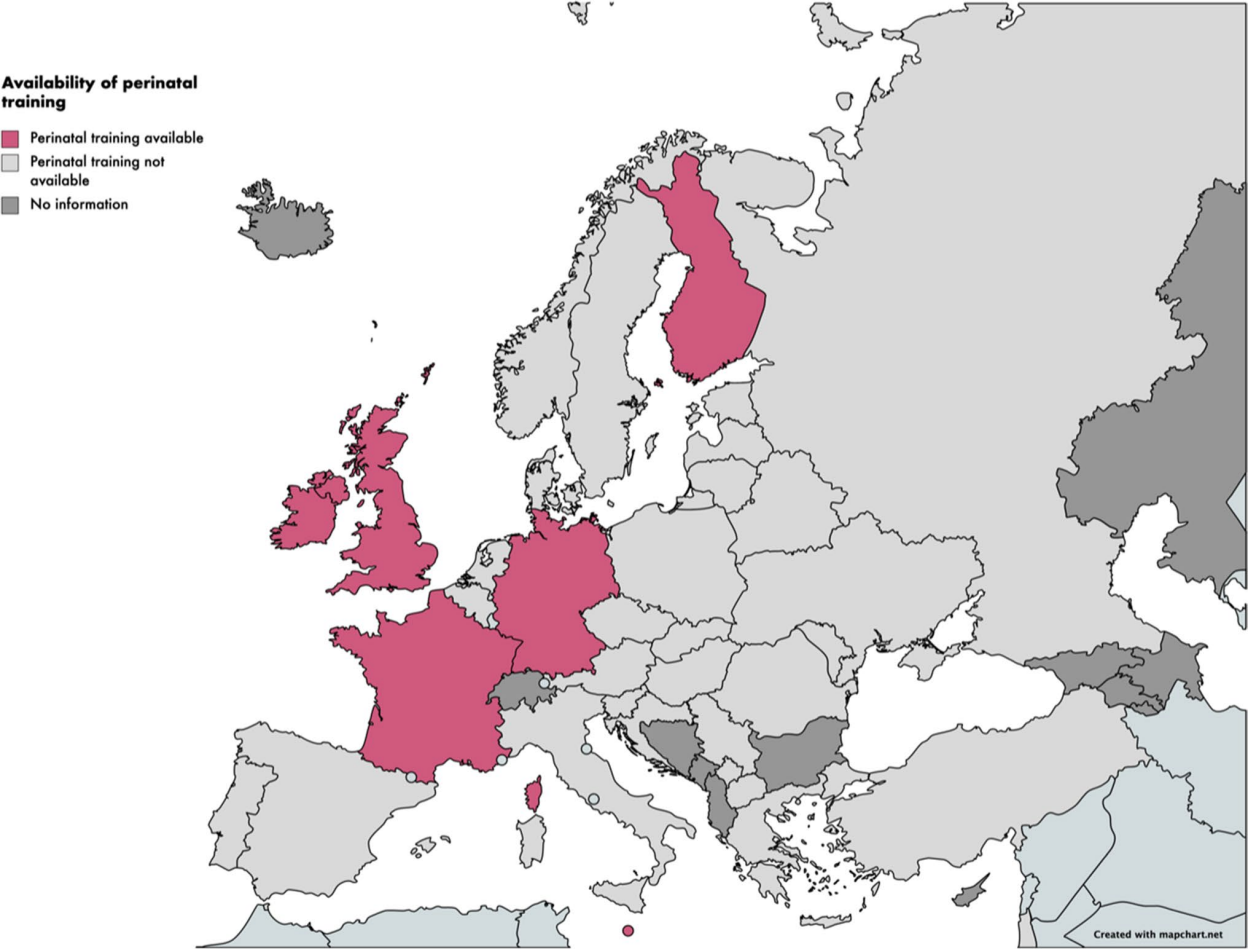
Map of European countries where specialist perinatal psychiatry training is available

Representatives for 34 of 37 (92%) EFPT member countries responded to the online survey. Six (6/34 = 18%) reported that training in perinatal psychiatry was available and participated in further in-depth interviews: Finland, France, Germany, Ireland, Malta, and the UK (see Fig. 1). Theoretical (didactic teaching) and practical training (in a clinical setting) were mandatory in only one country, Malta. The other five offered a mix of optional or mandatory theoretical and practical content in perinatal psychiatry (see Table 1).

**Table 1:** Description of perinatal psychiatric training

Country	Finland	France	Germany	Ireland	Malta	UK
Perinatal psychiatry training as part of	Child psychiatry	General adult psychiatry (as part of child psychiatry rotation)	General adult psychiatry	General adult psychiatry	General adult psychiatry	General adult psychiatry
Existing perinatal psychiatry training	Optional; Recommended for all CAP trainees	Optional	Optional	Mandatory theory; Optional practice	Mandatory	Mandatory theory; Optional practice
Type of training	Theory and practice	Theory and practice	Theory	Theory and practice	Theory and practice	Theory and practice
Length	1–12 months	6–12 months	Not specified	6–12 months	> 1 month	6–12 months
Setting of training	Young children’s unit/University hospital	Maternity wards	N/A	Maternity hospital	Special clinics	N/A
Supervision	Regular	Regular	N/A	Regular	N/A	Regular
Research opportunity	Yes	No	No	Yes	No	Yes
Assessment of training	N/A	N/A	N/A	N/A	N/A	Via Clinical Assessment of Skills and Competencies (CASC) exam
Length of psychiatric training	6 years	4 years	5 years	6 years	5 years	6 years
Training nationally standardised	No	Yes	No	Yes	Yes	Yes
Recognised sub-specialties	Yes	Yes	Yes	Yes	No	Yes
Liaison psychiatry training available	Yes	Yes	Yes	Yes	Yes	Yes

*Countries which do not offer perinatal psychiatry training as part of their regular curriculum: Austria, Belarus, Belgium, Croatia, Czech Republic, Denmark, Estonia, Greece, Hungary, Israel, Italy, Kosovo, Latvia, Lithuania, Macedonia, Moldova, Norway, Poland, Portugal, Romania, Russia, Serbia, Slovakia, Slovenia, Spain, Sweden, The Netherlands, Turkey, and Ukraine (N/A: not available)*

The duration of perinatal psychiatry training is highly variable (see Table 1), as is the training context. In Finland and France, it is provided as part of training in child psychiatry. All the other countries provide it as part of adult psychiatry training.

Clinical perinatal psychiatry training was reported to be organized within multidisciplinary teams composed of trainees, specialists, specialist nurses, child, and adult psychologists, as well as midwives, paediatricians, obstetricians, neonatologists, and geneticists. The most common clinical conditions assessed during clinical training were mood disorders and psychoses, but other cases included the psychological sequelae of obstetric/gynaecological complications such as miscarriages, high-risk pregnancies, preterm deliveries, and neonatal problems, including those requiring intensive care unit treatments.

### Experience of available perinatal psychiatry training

All interviewees (*n* = 6) consistently reported as a benefit of undertaking a rotation/training in perinatal psychiatry an increased competence in prescribing and managing psychopharmacological interventions during the perinatal period.

In addition, trainees (*n* = 3) reported the aforementioned multidisciplinary care model contributed to their professional development and confidence in managing a vulnerable population.

Moreover, interviewees (*n* = 3) highlighted that this training helped them appreciate the potential of the period as a window to effect a positive impact not only on a woman’s and family’s quality of life but on the development of an infant too.

### Reported preferences for specialist perinatal psychiatry training

Of the 28 countries which did not offer specialist perinatal psychiatry training as part of their regular curricula, the majority (22/28 = 76%) of respondents reported that it ought to be included in their country’s curriculum. Three (3/28 = 11%) did not, and four (4/28 = 14%) did not answer. Respondents not in favour of adding perinatal training to their curriculum (Belarus, Latvia, and Norway) cited lack of time in their training programme as the main barrier (1 year of postgraduate training in Belarus and four in Latvia).

Twelve (12/28 = 43%) country representatives expressed a preference for the addition of perinatal psychiatry training to their curricula as a mandatory component, while nine recommended it to be optional (9/28 = 32%) and seven (7/28 = 25%) did not answer. Sixteen representatives (16/28 = 57%) recommended both didactic and clinical training to be made available, four (4/28 = 14%) recommended didactic only, and eight (8/28 = 29%) did not answer.

The average duration of perinatal training suggested was 2 months.

## Discussion and conclusions

The current study is, to our knowledge, the first survey reporting the state of perinatal psychiatry postgraduate training in Europe from the perspective of trainee psychiatrists. With a response rate of 92% covering 34 European countries, it gives an overview of the characteristics of both the available and desired training in this important field.

As many as 1 in 5 women have pregnancies complicated by perinatal mental health problems (Howard et al. 2014; Jones et al. 2014). Although psychiatrists provide care throughout the lifespan, including to women in the perinatal period, we found that the specific training accessible to these doctors can be scarce and variable internationally. Although in many countries, a few hours of teaching may be spent on psychiatric problems and psychopharmacology in the perinatal period, only 6 out of 34 surveyed European countries explicitly provide specialist perinatal psychiatry training of any modality and length. This mirrors the deficit of specialist perinatal services across Europe (Brockington et al. 2017; Howard and Khalifeh 2020). There is no official source of aggregate data about perinatal psychiatry provision across Europe. The UK, France, Switzerland, Austria, Belgium, Germany, Israel, and the Netherlands have mother-baby units but, in many cases, less than 1 million population (Brockington et al. 2017). The knowledge of the state of perinatal psychiatry training in other areas of the world is scarce, despite knowing that some countries, e.g. India, do have mother-baby units. In the US, a survey of trainees and trainers showed great inconsistency, and the authors suggested—and later developed—a standardised curriculum to be used across the different states (Osborne et al. 2018). If the services and the specialists do not exist, it becomes harder to provide specialist training. An additional benefit of developing specialist perinatal psychiatry providers includes improved access to training for allied maternity healthcare providers. This would ensure the delivery of cross-discipline mental healthcare-informed treatment.

Trainees highlighted the psychopharmacology skills they gained as beneficial and important in the management of women in the perinatal period. This may also reflect that during training, more emphasis is placed on pharmacology rather than psychotherapy (Gargot et al. 2017).

The main limitation of our study is that it assumes the information provided by individual participants is an authoritative account of national training infrastructure. These individuals are, however, well placed to answer these questions as they are national trainee representatives with access to the most current postgraduate training information, in addition to their first-hand experience as trainees. For countries without centralised and standardised training, it is more difficult to profile training at a national level. We cannot, therefore, comment on the possibility of local training centres offering perinatal psychiatry training to a limited number of trainees. Our results are not a commentary on whether such training currently *exists* but whether it is *accessible* to trainees.

Comprehensive psychiatric healthcare will always include the care of women of childbearing age, many of whom will become pregnant. Our recommendation is that core curricula and standards of training must include sufficient knowledge and clinical competencies to care for this population group. This would ensure a universal level of mental healthcare in the perinatal period. In addition to didactic teaching, there should also be at least an optional rotation in a relevant clinical service (e.g. perinatal, liaison, etc.). Solely relying on ‘over-specialized’ psychiatrists will never be sufficient to meet clinical needs, and we must ensure general psychiatrists have the relevant skills. If not acquired during training, competencies could be developed through continuous medical education: online or face-to-face courses, post-specialty credentials, and/or exchange/shadowing programs. UEMS and EFPT have a role to play by updating their training recommendations to include perinatal psychiatry training. Other organisations devoted to perinatal mental health can contribute with expert topic knowledge. Further research can focus on exploring trainees’ and trainers’ experiences and further define the standard criteria for specialty training in perinatal psychiatry and what a high-quality placement would look like.

In conclusion, perinatal mental health training requires much greater attention than it receives today. The population consequences of perinatal psychiatric morbidity are vast and extend even beyond the women affected to include fathers, partners, and infants. Therefore, the widespread deficiency of appropriate training profiled in this study should serve as a wake-up call for clinicians, training bodies, and policymakers alike.

## Electronic supplementary material

ESM 1 (DOCX 18.7 kb)

## Data Availability

Available upon reasonable request.

## Code availability

N/A
