# Supplementary material for: Psychiatric training in perinatal mental health across Europe
Source: Arch Womens Ment Health. 2022 Mar 3;25(2):501–6. doi: 10.1007/s00737-022-01216-w (PMC8921130; doi:10.1007/s00737-022-01216-w)
Supplement: Supplementary file 1 — (DOCX 18.7 kb) [file 737_2022_1216_MOESM1_ESM.docx]

Supplement

Methods

*Survey respondents*

The survey was sent to representatives of each national association of trainee psychiatrists, who replied in their representative capacity. One report was completed per country, even where there was more than one national association e.g. certain countries have a national association for trainees in adult psychiatry and another for trainees in child and adolescent psychiatry, where those specialties are separate.

In countries with established national committees representing trainees, the respondent was generally a prominent member of that committee with access to accurate and comprehensive national training data. Where there was no official national association, as either an association is yet to be officially established or no longer exists, trainees with comparable knowledge were contacted. They were chosen because they have similar positions of responsibility and in-depth knowledge of the training structure and content within their countries, for example former national trainee representatives, or individuals engaged in other national professional networks such as Early Career Psychiatrists’ representatives for the European or World Psychiatric Associations.

*Survey questionnaire*

This survey was part of a broader online survey of postgraduate training which EFPT conducts annually of all member countries. The online questionnaire consisted of general questions about postgraduate psychiatry training and specific questions related to perinatal psychiatry.

General questions about training:

- What is the total duration of psychiatry training in your country? (please specify in years)?
- Is training in your country nationally standardised?
- Are there any recognised subspecialties of Psychiatry in your country?
- Please specify if a rotation in liaison psychiatry is available, mandatory and how long is the duration of the rotation is in your country?

Specific questions about perinatal training:

- Do you have training in perinatal mental health?
- Please describe your training in perinatal mental health. If you don’t have any, how do you think it should be organised?
- Mandatory or optional?
- Theory or practice?
- Duration?

We clarified any unclear answers with the representatives.
